# Identification of a CD4+ conventional T cells-related lncRNAs signature associated with hepatocellular carcinoma prognosis, therapy, and tumor microenvironment

**DOI:** 10.3389/fimmu.2022.1111246

**Published:** 2023-01-09

**Authors:** Lin Zhu, Xiu-Ping Zhang, Shuai Xu, Ming-Gen Hu, Zhi-Ming Zhao, Guo-Dong Zhao, Zhao-Hui Xiao, Rong Liu

**Affiliations:** ^1^ Medical School of Chinese PLA, Beijing, China; ^2^ Faculty of Hepato-Biliary-Pancreatic Surgery, the First Medical Centre, Chinese People’s Liberation Army (PLA) General Hospital, Beijing, China; ^3^ Institute of Hepatobiliary Surgery of Chinese PLA, Beijing, China; ^4^ Key Laboratory of Digital Hepatobiliary Surgery, PLA, Beijing, China; ^5^ The First Clinical Medical School, Lanzhou University, Lanzhou, China; ^6^ Department of Liver Transplantation and Hepatobiliary Surgery, Shandong Provincial Hospital Affiliated to Shandong First Medical University, Jinan, China

**Keywords:** CD4+ conventional T cells, long non-coding RNA, hepatocellular carcinoma, tumor microenvironment, prognostic signature

## Abstract

**Background:**

Hepatocellular carcinoma (HCC) is the second leading cause of cancer-related death worldwide, and CD4+ T lymphocytes can inhibit hepatocarcinogenesis and mediate tumor regression. However, few studies have focused on the prognostic power of CD4+ Tconv-related lncRNAs in HCC patients.

**Method:**

We obtained data from TCGA and GEO databases and identified CD4+Tconv-related lncRNAs in HCC. The risk score was constructed using lasso regression and the model was validated using two validation cohorts. The RS was also assessed in different clinical subgroups, and a nomogram was established to further predict the patients’ outcomes. Furthermore, we estimated the immune cell infiltration and cancer-associated fibroblasts (CAFs) through TIMER databases and assessed the role of RS in immune checkpoint inhibitors response.

**Results:**

We constructed a CD4+ Tconv-related lncRNAs risk score, including six lncRNAs (AC012073.1, AL031985.3, LINC01060, MKLN1-AS, MSC-AS1, and TMCC1-AS1), and the RS had good predictive ability in validation cohorts and most clinical subgroups. The RS and the T stage were included in the nomogram with optimum prediction and the model had comparable OS prediction power compared to the AJCC. Patients in the high-risk group had a poor immune response phenotype, with high infiltrations of macrophages, CAFs, and low infiltrations of NK cells. Immunotherapy and chemotherapy response analysis indicated that low-risk group patients had good reactions to immune checkpoint inhibitors.

**Conclusion:**

We constructed and validated a novel CD4+ Tconv-related lncRNAs RS, with the potential predictive value of HCC patients’ survival and immunotherapy response.

## 1 Background

Hepatocellular carcinoma (HCC) is the second leading cause of cancer-related death with an increasing incidence worldwide (1). Risk factors of HCC pathogenesis include hepatitis virus infection, alcohol, non-alcohol fatty liver disease (NAFLD), and aflatoxin B1 intake (2). Research on the prevention and treatment of HCC continues to receive attention. According to 2022 Barcelona Clinic Liver Cancer (BCLC) recommendations, HCC is primarily treated by surgery (resection and transplant) and/or in combination with multiple adjuvant therapies, such as ablation, transarterial chemoembolization (TACE), and systemic treatment (3–8). These treatments, combined with molecular biomarkers like alpha-fetoprotein, have reduced liver cancer-related deaths to a certain extent, but the prognosis of HCC patients remains unsatisfactory. Therefore, it is necessary to find novel effective molecular biomarkers to guide clinical treatment and improve outcomes for HCC patients.

Early studies focused more on protein-coding genes because of their crucial roles in the biological process of HCC and noncoding RNAs were often ignored. However, in recent years, increasing evidence has confirmed that long noncoding RNAs (lncRNAs) play important roles in the pathological process, including HCC (9). LncRNAs played various roles in the HCC process. Previous studies showed that lncRNA HULC was upregulated in HCC and promoted proliferation, metastasis, and drug resistance of HCC (10, 11). While lncRNA-XIST could inhibit the growth and metastasis of HCC by interacting with miR-92b (12). LncRNAs also played key roles in regulating tumor microenvironment (TME) and tumor immune microenvironment (TIME). Up-regulating lncRNAs, like lncRNA-IFI6 and EGOT, could inhibit the expression of IFN-stimulated genes (ISGs) and lead to immunosuppression (13, 14). In addition, some HCC-related lncRNAs exist in body fluids that are easy to detect and analyze, thus they have the potential to become novel biomarkers for HCC patients (9).

Previous studies indicated that CD4+ T lymphocytes could inhibit hepatocarcinogenesis and mediate tumor regression (15, 16). In addition, some non-synonymous cancer mutations were immunogenic, and most immunogenic mutations could be recognized by CD4+ T lymphocytes (17). Deletion of CD4+ T lymphocytes led to the development of HCC (18). These findings demonstrated that CD4+ T conventional lymphocytes (CD4+ Tconv) played a key role in HCC. However, due to the limitations of sequencing technology, few studies focused on the prognostic ability of CD4+ Tconv-related lncRNAs in HCC patients.

With the development of single-cell RNA sequencing (scRNA-seq) technology, researchers can obtain more data about liver cancer and study HCC in greater depth. In this way, bioinformatic analysis can be used to process the accumulating scRNA-seq data, which can help us identify the key lncRNAs, predict the prognosis of HCC patients and learn more about TME and TIME of HCC. This study aimed to establish a CD4+ Tconv-related lncRNAs prognostic signature to predict the prognosis and systemic treatment effect of HCC patients. Based on scRNA-seq data and gene expression data, we constructed a CD4+ Tconv-related lncRNAs prognostic signature. We evaluated the signature efficacy in different clinical subgroups and assessed the relationship between risk signature and TME. Furthermore, we analyzed the systematic treatment efficacy in different risk groups, hoping to find more potential applications of our novel risk signature in clinical treatment.

## 2 Methods

### 2.1 Data collection

Gene expression data and corresponding clinical data for HCC were downloaded from The Cancer Genome Atlas (TCGA, https://portal.gdc.cancer.gov/). Patients without survival information were excluded. HCC scRNA-seq data (GSE140228) in both platforms (SMART-seq2 and droplet-based platforms) were obtained from the Gene Expression Omnibus database (GEO, https://www.ncbi.nlm.nih.gov/geo/) (19).

### 2.2 Identification of CD4+Tconv-related lncRNAs in HCC

The Tumor Immune Single-cell Hub (TISCH) is a scRNA-seq database focusing on TME (20). We used TISCH to extract CD4+ Tconv-related genes from HCC scRNA-seq data. Pearson correlation was applied to calculate the correlation between lncRNAs and CD4+ Tconv-related genes. LncRNAs with absolute correlation coefficients > 0.5 and p <0.001 were considered CD4+Tconv-related lncRNAs (21). Co-expression networks were visualized using Cytoscape software (22).

### 2.3 Construction of CD4+Tconv-related lncRNAs risk score

The least absolute shrinkage and selection operator (LASSO) regression was performed to identify the optimal prognostic lncRNAs with 10-fold cross-validation (23). Univariate Cox regression was performed to further evaluate the LASSO results, and finally, six CD4+Tconv-related lncRNAs significantly associated with overall survival (OS) were selected. Based on these lncRNAs, a CD4+Tconv-related lncRNAs risk score (RS) was established using multivariate Cox regression. All patients were divided into the high-risk group and low-risk group based on the median RS, and Kaplan-Meier (K-M) survival analyses were performed to estimate OS between the two groups. Time-dependent receiver operating characteristic (ROC) curve analysis was used to assess the prognostic value of RS over time. In addition, all HCC patients in TCGA were randomly assigned into validation 1 and validation 2 groups according to a ratio of 1:1. K-M survival analysis and time-dependent ROC curve analysis were also performed in both validation cohorts.

To further understand the clinical relevance of the RS, we explored the role of the RS in different clinical subgroups (age, sex, American Joint Committee on Cancer (AJCC) stage, TNM stage, prior malignancy, pharmaceutical therapy, and radiation therapy). We assessed the Hazard Ratio (HR) for RS in different subgroups and used the forest plot to demonstrate the results.

Based on the risk score and other clinical data, we constructed a nomogram using “survival” and “rms” packages. The concordance index (C-index) and calibration curves were used to assess the accuracy and predictive ability of the model. In addition, Decision Curve Analysis (DCA) was used to evaluate and compare the clinical utility between the nomogram and AJCC stage.

### 2.4 Functional enrichment analysis

The “clusterProfiler” R package was used to perform Gene Ontology (GO) and Kyoto Encyclopedia of Genes and Genomes (KEGG) pathway enrichment analyses (24). The difference between high-risk group and low-risk groups were estimated by Gene Set Enrichment Analysis (GSEA) algorithm (25). The gene set file for functional enrichment analysis was obtained from the MSigDB database (https://www.gsea-msigdb.org).

### 2.5 Tumor Microenvironment Analysis and Immunotherapeutic Effect

The Tumor Immune microenvironment analysis and immunophenoscore Estimation Resource (TIMER) database is a comprehensive resource for systematical analysis of immune filtrates and we use TIMER 2.0 to estimate the immune cell infiltrations between high-risk and low-risk groups (26). The “ESTIMATE” package was used to assess the immune scores for different risk groups (27). Meanwhile, immune checkpoint genes were used to estimate the immunotherapeutic sensitivity between high-risk and low-risk groups (28), and immunophenoscore obtained from The Cancer Group Atlas (TCIA, https://tcia.at/home) was used to predict the response to checkpoint blockade (29). Tumor mutation burden (TMB) data was obtained from the TCGA database and the “maftools” R package was used to analyze the TMB.

### 2.6 Statistical analysis

All statistical analyses were performed using R version 4.1.1. Differences between the two risk groups were assessed using the Mann-Witney-Wilcoxon test. Survival analysis was performed using the R packages “survival” and “survminer”. P<0.05 was considered statistically significant. The median value of continuous variables was recognized as the cutoff value.

## 3 Results

### 3.1 Analysis of liver cancer single-cell sequencing data

**Figure 1:**
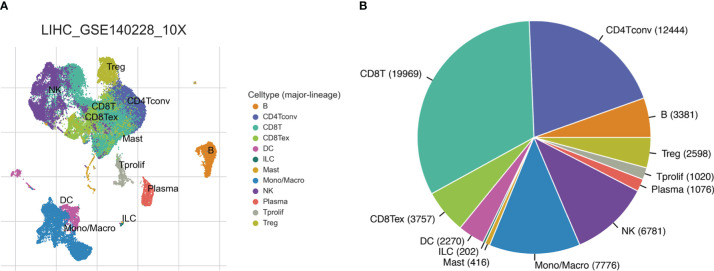
HCC single-cell sequencing data from the droplet-based platform. **(A)** The UMAP plot of different cell types. **(B)** Ratios of different cell types.

The workflow of this study was shown in **Supplementary Figure 1**. We obtained single-cell sequencing (scRNA-seq) data from GSE140228 and analyzed the immune microenvironment of hepatocellular carcinoma based on the TISCH database. GSE140228 provides scRNA-seq data based on two platforms (SMART-seq2 and droplet-based platforms). We analyzed and visualized the major cell type of liver cancer, with conventional CD4+ T cells accounting for a large proportion of the HCC immune microenvironment. The HCC cancer scRNA-seq data analysis of GSE140228 in droplet-based and SMART-seq2 platforms were shown in **Figure 1** and **Supplementary Figure 2** respectively.

### 3.2 Identification and functional enrichment of CD4+ Tconv-related genes and lncRNAs

**Figure 2:**
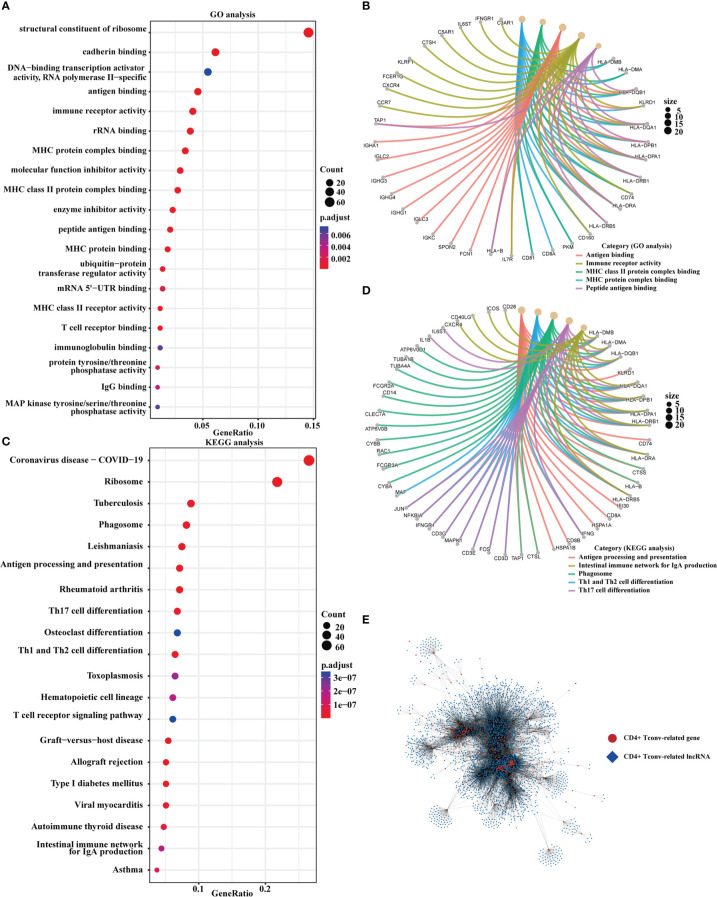
Functional enrichment and the co-expression network of CD4+ Tconv-related genes. **(A, B)** The dot plot and the network graph of GO analysis. **(C, D)** The dot plot and the network graph of the KEGG pathways analysis. **(E)** Co-expression network of CD4+ Tconv-related genes and lncRNAs.

We obtained CD4+ Tconv-related genes from GSE140228 in two platforms and identified 478 intersect CD4+ Tconv-related genes. Based on the intersecting CD4+ Tconv-related genes, the molecular mechanisms of these genes were clarified through the GO and KEGG pathway enrichment analysis. The enrichment analysis indicated the significant enrichment of immune-related functions and pathways, like MHC protein complex binding, MHC class II protein complex binding, T cell receptor binding, antigen processing and presentation, Th1 and Th2 cell differentiation, Th17 cell differentiation, natural killer cell mediated cytotoxicity, etc. We used network graphs to further demonstrate the relationship between selective GO and KEGG pathways results. Based on GSE140228 and TCGA data, we built a CD4+ Tconv-related gene and lncRNA co-expression network and identified 3877 CD4+ Tconv-related lncRNAs. The functional enrichment results and co-expression network were shown in **Figure 2**.

### 3.3 The construction and validation of CD4+ Tconv-related lncRNAs prognosis signature for HCC

We obtained the CD4+ Tconv-related genes from scRNA-seq data of HCC and identify the CD4+ Tconv-related lncRNAs for further research. Based on TCGA-LIHC data, we extracted survival-related lncRNAs and used the lasso method and cox regression to construct an HCC prognostic risk signature score model. The lasso regression analysis was shown in **Supplementary Figure 3**. The risk signature finally contained six lncRNAs and the risk score (RS) was calculated as follows: RS = (0.14741432*AC012073.1 exp.) + (0.07721218*AL031985.3 exp.) + (0.74153487*LINC01060 exp.) + (0.25333105*MKLN1-AS exp.) + (0.07475760*MSC-AS1 exp.) + (0.08527743*TMCC1-AS1 exp.). According to the median RS, all patients were divided into high-risk and low-risk groups. The Kaplan-Meier curve analysis demonstrated that patients in the high-risk group had a worse prognosis (p<0.0001). Time-dependent ROC analysis was used to evaluate the predictive ability of the model, and the AUC values and 95% confidence interval (CI) at 1, 3, and 5 years for predicting OS were 0.71 (0.65-0.76), 0.66 (0.59-0.72), 0.67 (0.58-0.75) respectively.

**Figure 3:**
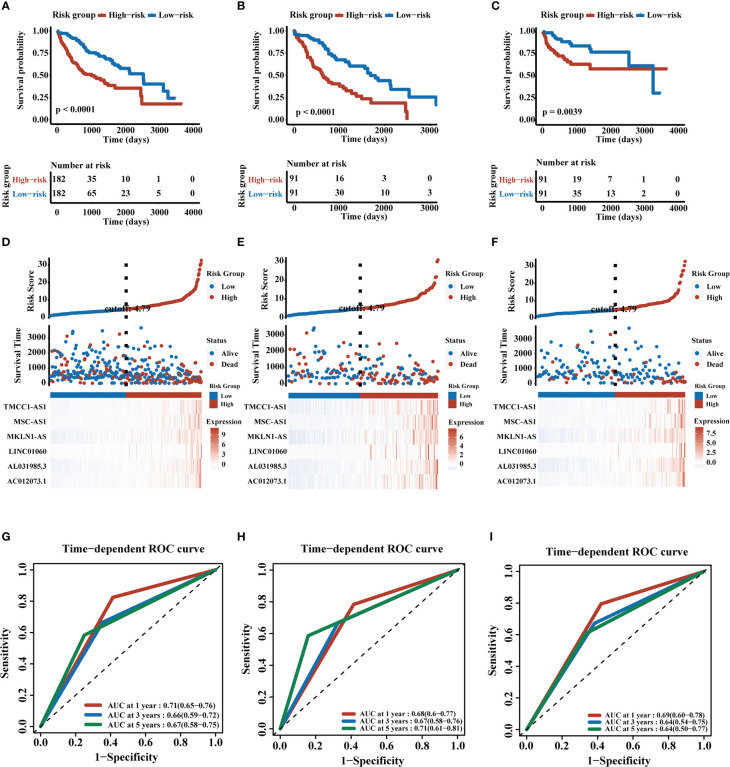
Construction and validation of CD4+ Tconv-related lncRNAs prognosis signature. **(A–C)** K-M plots of training cohort, validation cohort 1, and validation cohort 2. **(D–F)** RS distribution, survival status, and heatmap of expression of six CD4+ Tconv-related lncRNAs in high-risk and low-risk groups in three cohorts. **(G–I)** Time-dependent ROC analysis of three cohorts.

TCGA-LIHC patients were randomly divided into two groups in a 1:1 ratio, and we used these two groups as validation cohorts for internal validation. The results showed that the high-risk group had a poorer prognosis in both validation cohorts, all six lncRNAs were highly expressed in the high-risk group, and time-dependent ROC analysis further confirmed the stability of the RS model (Validation cohort 1, AUC and 95% CI at 1, 3, and 5 years for predicting OS were 0.68 (0.60-0.77), 0.67 (0.58-0.76), and 0.71 (0.61-0.81) respectively; Validation cohort 2, AUC and 95% CI at 1, 3, and 5 years for predicting OS were 0.69 (0.60-0.78), 0.64 (0.54-0.75), 0.64 (0.50-0.77) respectively). We also demonstrated the trend of AUC over time in three cohorts and we found that the AUC value is relatively stable over time. The K-M curves, distribution of risk score, survival status and lncRNAs expression, and time-dependent ROC results were shown in **Figure 3** and **Supplementary Figure 4**. We analyzed the demographic data of both groups and the results showed that both groups had similarly representative clinical factors. The outcomes were shown in **Supplementary Table 1**.

### 3.4 Identifying the role of RS in clinical subgroups and establishing a prognostic nomogram

**Figure 4:**
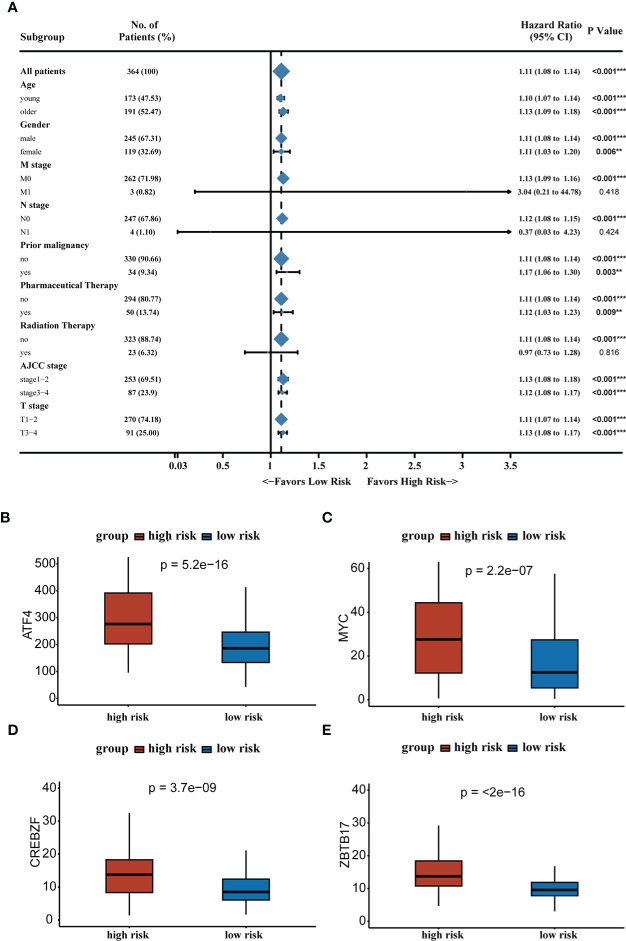
**(A)** Forest plot demonstrating the role of RS in different clinical subgroups. **(B–E)** Expression levels of vascular invasion-related genes in high-risk and low-risk groups. **P < 0.01, ***P < 0.001.

We analyzed RS in patients with different clinical characteristics (including age, gender, AJCC stage, T stage, N stage, M stage, prior malignancy, pharmaceutical therapy, and radiation therapy) to further develop the application value of RS and test its accuracy. The forest plot demonstrated that the high RS was a poor prognostic factor in most clinical subgroups except for patients in the N1 stage, M1 stage, and receiving radiation therapy subgroups. The forest plot was shown in **Figure 4A**. Microvascular invasion was one of the most important prognostic factors in HCC, and it was necessary to estimate the relationship between RS and microvascular invasion. According to the previous study, MYC, CREBZF, HOXD13, and ZBTB17 were the most significant upstream regulators of the vascular invasion-related transcriptome (30). We compared the gene expression between two groups and high-risk group patients had higher expression levels of vascular invasion-related genes, which partly contributed to a poor prognosis. The results were shown in **Figures 4B–E**.

**Figure 5:**
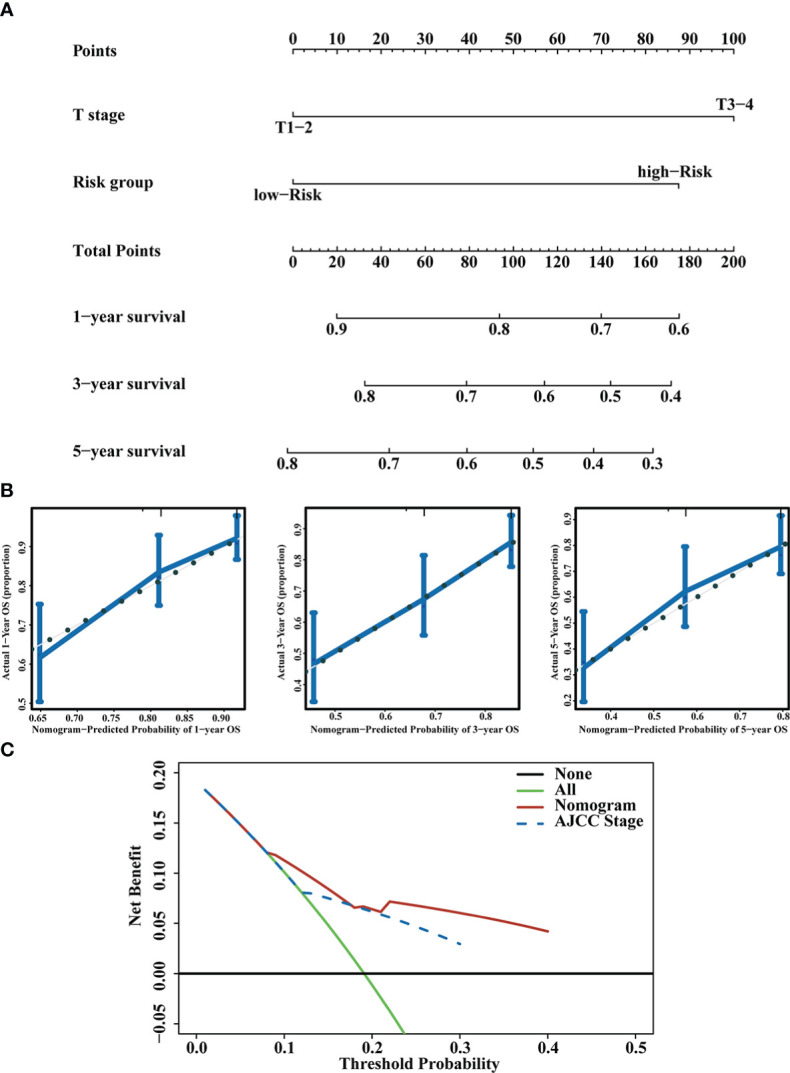
Prognostic nomogram construction. **(A)** Nomogram integrating the RS and T stage. **(B)** Calibration curves for predicting 1,3 and 5 years OS. **(C)** DCA of the nomogram and AJCC stage.

To improve the clinical utility of the CD4+ Tconv-related lncRNAs model, we constructed a nomogram to predict the OS of patients. Each patient had an integrated point based on their AJCC T stage and RS group. Using the integrated points, we can predict the 1, 3, and 5 years survival rates of patients. The concordance index (C-index) and 95% CI of the nomogram was 0.685 (0.651-0.719), indicating that the nomogram had a good ability to distinguish between high-risk and low-risk patients. Calibration curves were used to test the consistency between the predicted risk and actual risk of the prediction model, and the results showed that the OS predicted by the nomogram was close to the actual OS probability. Furthermore, we used DCA to compare the differences between our risk model and the AJCC stage, and the DCA showed that, in most cases, the risk model was not inferior to the AJCC stage in predicting OS of HCC patients. The nomogram, calibration curves, and DCA were shown in **Figure 5**.

### 3.5 The relationship between RS and tumor microenvironment

**Figure 6:**
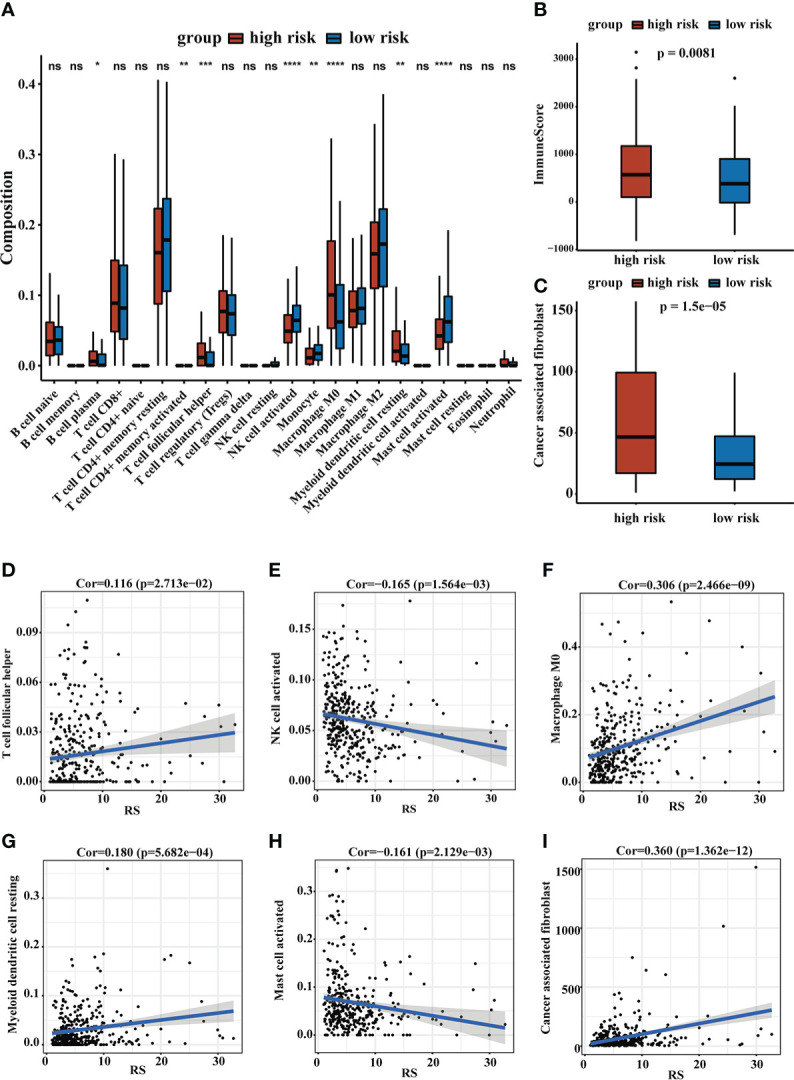
The landscape of TME. **(A)** The relative cellular abundance of immune cells was calculated by CIBERSORT. **(B)** The ImmuneScore was calculated by ESTIMATE. **(C)** The relative infiltration degree of CAFs between the two groups. **(D–I)** The correlation analysis of immune infiltrating cells and RS. ns, no significance, *P < 0.05, **P < 0.01, ***P < 0.001, ****P < 0.0001.

The TME plays an important role in the occurrence and development of liver cancer, so it is necessary to evaluate the relationship between RS and TME. We calculated immune scores in HCC patients using the ESTIMATE method, and we found that high-risk group patients tended to have higher immune scores. To further explore the TIME, we use the TIMER database and the MCPcounter method to estimate the cell infiltration of HCC patients. The high-risk group had a higher proportion of immune cell infiltration in B cell plasma, T cell follicular helper, macrophage M0 and myeloid dendritic cell resting, while the low-risk group had a higher infiltration rate of activated natural killer (NK) cells and activated mast cells. The MCPcounter result indicated that the high-risk group tended to have higher MCPcounter scores in CAFs. Correlation analyses of immune infiltration cells and RS achieved similar results. The results were shown in **Figure 6**.

**Figure 7:**
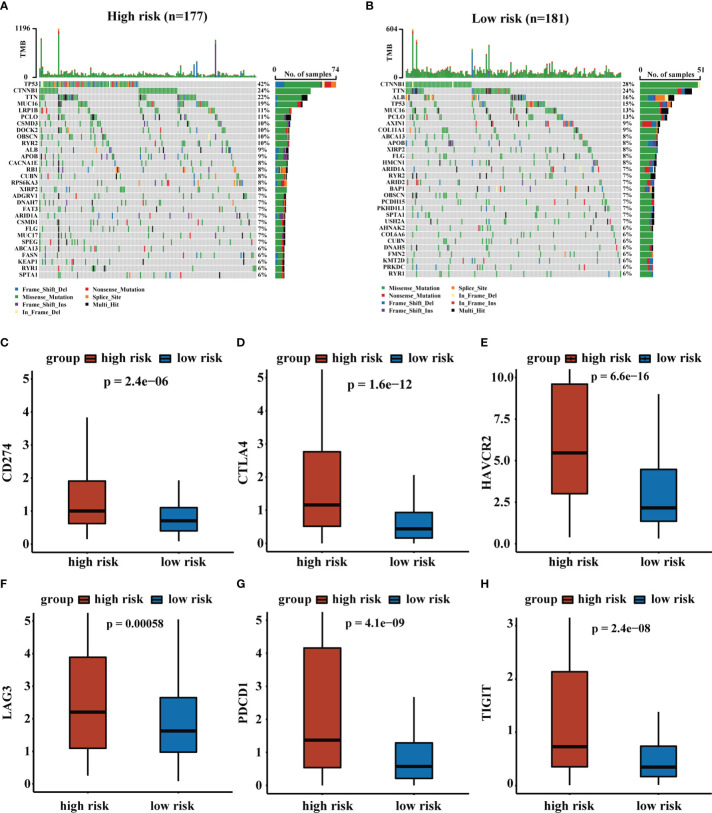
The analysis of TMB and expression levels of immune checkpoints genes. **(A, B)** Top 30 gene mutations in high-risk and low-risk groups. **(C–H)** Expression levels of immune checkpoints genes in high-risk and low-risk groups.

We assessed the top 30 mutated genes in both risk groups. Furthermore, we determined several checkpoint genes (CD274, CTLA4, HAVCR2, LAG3, PDCD1, and TIGIT) and found that most checkpoint genes had higher expression in the high-risk group (31). The oncoplot of TMB and expression levels of checkpoint genes were shown in **Figure 7**.

### 3.6 Evaluation of response to immunotherapy and chemotherapy in patients with liver cancer

**Figure 8:**
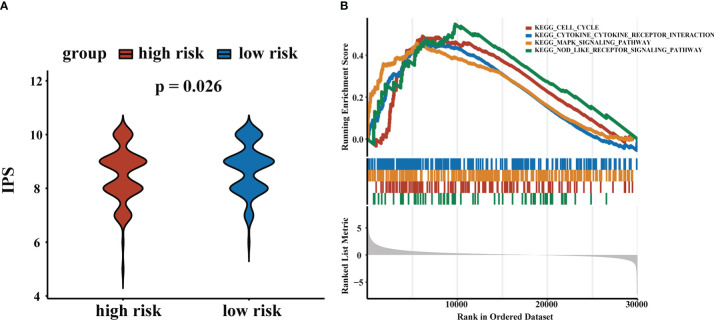
Application of RS in chemotherapy and immunotherapy. **(A)** IPS scores for high-risk and low-risk groups. **(B)** GSEA results.

The CD4+ Tconv-related lncRNA RS were closely related to TME and time in HCC patients. To explore the role of RS in response to immunotherapy, we obtained immunophenoscore (IPS) from the TCIA database and found that patients in the low-risk group tended to have higher IPS, suggesting that patients in the low-risk group were more sensitive to immunotherapy. The results demonstrated that our RS may contribute to the clinical treatment of HCC patients. We performed GSEA to further evaluate functional pathways that were significantly enriched in high-risk and low-risk groups. The results indicated that these pathways were related to cell cycle, MAPK signaling pathway, cytokine-cytokine receptor interaction, and NOD-like receptor signaling pathway. The results were shown in **Figure 8**.

## 4 Discussion

HCC, the most common primary liver cancer, is a common solid tumor with a high recurrence rate and poor prognosis (32, 33). Systemic therapy is recognized as a crucial management component for HCC patients and a possible combination of immune checkpoint inhibitors (ICIs) is a promising approach for the treatment of HCC patients (34). However, the majority of patients do not respond to immunotherapy and some may deteriorate and develop hyperprogressive diseases (35, 36). CD4+ Tconv plays an important role in the antitumor response and can enhance patient immunity by secreting cytokines and activating CD8+ T lymphocytes (37, 38). Furthermore, LncRNAs are important prognostic markers for predicting the prognosis of HCC patients (39). Therefore, it is essential to identify the biomarkers of CD4+ Tconv-related lncRNAs that can guide patient-specific therapy choices (34). In this study, we conducted a comprehensive analysis of the prognostic value of CD4+ conventional T cells-related lncRNAs in hepatocellular carcinoma based on single-cell RNA sequencing data. We constructed a CD4+ Tconv-related lncRNAs risk score with good predictive power in validation cohorts and most clinical subgroups. We also constructed a nomogram with comparable OS predictive power compared to the AJCC staging system. Our risk score can also predict the immune cell infiltrations of the tumor immune microenvironment and response to immune checkpoint inhibitors.

In our research, we obtained the scRNA-seq data from GSE140228 and identified the CD4+ Tconv-related genes through the TISCH database. We demonstrated CD4+ Tconv-related lncRNAs using a co-expression network. Through Cox regression and LASSO algorithm, six CD4+ Tconv-related lncRNAs (AC012073.1, AL031985.3, LINC01060, MKLN1-AS, MSC-AS1, TMCC1-AS1) were identified, and used to construct a prognostic risk model. All these lincRNAs played essential roles in HCC biological process. Studies showed that lncRNA AC012073.1 played an oncogenic role in influencing HCC prognosis and was associated with N6-methyladenosine and ferroptosis (40, 41). AL031985.3 was associated with pyroptosis, immune response, ferroptosis, glycolysis, and autophagy in HCC (42–45). LINC01060 was aberrantly expressed in various cancers, including glioma and pancreatic cancer, and was significantly associated with poor clinical prognosis (46, 47). MSC-AS1 functioned as a key oncogene by inducing cell growth, cell cycle progression, and inflammatory mediators secretion (48). TMCC1-AS1 predicted poor prognosis and accelerated epithelial-mesenchymal transition in liver cancer (49). MKLN1-AS increased HDGF expression by acting as a molecular sponge for miR-654-3p, inducing pro-oncogenic effects during HCC progression (50). All these lincRNAs were highly expressed in the high-risk group. The accuracy and stability of the risk model were validated in Validation Cohort 1 and Validation Cohort 2, and the forest plot was used to explore the role of the risk model in different clinical subgroups. RS was a risk factor for most clinical subgroups, except in patients with N1 stage or M1 stage tumors or those receiving radiation therapy. We further developed a nomogram model containing risk score and T stage, used calibration curves to assess the predictive power of the nomogram, and compared the effect between the nomogram model and the AJCC stage. Our nomogram had good predictive power of OS, especially for 3-year OS, and it also had the comparable predictive ability of OS compared with the AJCC stage.

Furthermore, TME plays an essential role in tumor development. Recent clinical trials have demonstrated that immune cell infiltration of TME *in situ* is considered a valuable indicator of the prognosis and immunotherapy response of cancers (51). HCC processed a rather intricate TME, which contained abnormal extracellular matrix, immune cells, CAFs, tumor-associated endothelial cells or macrophages, and their products (52, 53). Cell-to-cell interactions within the TME, as well as genomic and epigenomic alterations, greatly contribute to tumor heterogeneity and determine its malignant nature including prognosis and drug resistance (52). We used multiple computational methods to assess the abundance of immune cells and other cancer-related cell infiltration to reveal TME in HCC patients. Our results showed that plasma cells, follicular helper T (Tfh) cells, macrophage M0 and myeloid dendritic cell resting were more enriched in the high-risk group, while activated NK cells and activated mast cells were more enriched in the low-risk group. ScRNA-seq results showed that B cells, predominantly plasma cells, were more enriched in HCC-infiltrating immune cells compared with the cirrhotic and healthy liver, and HCC patients with a low proportion of tumor-infiltrating plasma cells presented a better prognosis (54). Tfh cells were a subset of CD4+ T cells and were essential for the germinal center formation and the development of most high-affinity antibodies and memory B cells (55). NK cells can respond to cell-surface ligands and release cytokines (53). Tumor-associated macrophages could promote the expansion of human hepatocellular carcinoma stem cells by producing IL6 (56). Dendritic cells (DCs) were the most potent antigen-presenting cells (57). and our results suggested a higher infiltration rate of resting myeloid DCs in the high-risk group, which may contribute to a poorer immune response. The data from the ESTIMATE method showed that the high-risk group had a higher immune score, which could represent the higher levels of immune cell infiltration in the TME and was consistent with previous studies (58, 59). However, our study showed that high risk patients had poor immunotherapy response and high immune cell infiltration levels did not mean a better immunotherapy response. Exhausted or dysfunctional T cells may partly contribute to the results. Moreover, experiments and sequencing data were required to further explain the outcomes. In addition, the MCPcounter results indicated the high-risk group was associated with higher infiltration levels of CAFs. CAFs can maintain and enhance the stemness of HCC, including proliferation, self-renewal, migration, invasion, drug resistance, tumorigenesis, and metastasis of HCC cells (60). In summary, we inferred that the poor prognosis of high-risk group patients may be related to this tumor immunosuppressive microenvironment and high infiltration levels of CAFs.

In addition, we found that the expression levels of immune checkpoint genes were higher in the high-risk group. Negative regulation of immune checkpoint molecules was one of the major factors leading to the exhaustion or dysfunction of effector memory CD8+ T cell, and late exhausted or dysfunctional T cells, representing impaired proliferation ability and reduced production of cytokines, further led to a weakened anti-tumor response (61). High expression levels of TIGIT and PDCD1 were recognized as immunosuppressed tumors with the poorest prognosis (62). Meanwhile, we further predicted the response to immunotherapy in high- and low-risk groups of HCC patients using IPS, with patients in the low-risk group associated with increased immunogenicity. These results suggested that the RS was a potential predictor of immunotherapy response, which meant that RS could provide a reference for clinical practice.

Currently, many studies are focused on precision genomic medicine, and many predictive models have been developed in an attempt to predict patient outcomes. Many lncRNAs, like N6-methyladenosine-related lncRNAs, ferroptosis-related lncRNAs, pyroptosis-related lncRNAs, glycolysis-Related lncRNAs, contributed to a better understanding of HCC biological processes and helped predict the prognosis of HCC (40–45). However, few studies concentrated on CD4+ Tconv-related lncRNAs, and the roles of CD4+ Tconv-related lncRNAs remained unclear. It is generally believed that the complex heterogeneity greatly hinders the therapeutic effect of molecularly targeted drugs in patients with advanced HCC. The advent of scRNA-seq has contributed to a better understanding of tumor heterogeneity in malignant cells at the transcriptomic level (52). Furthermore, the droplet-based platform captures large-scale cells at the expense of limited gene coverage, whereas SMART-seq2 provides deep coverage for smaller numbers of cells (63). The combination of these two techniques can enable an in-depth understanding of the TME landscape (19). We constructed a CD4+ Tconv-related lncRNAs RS to predict the prognosis of patients and hoped to provide references for clinical practice.

Our study had some limitations. First, the data were based on public databases and the RS should be further validated through large-scale prospective studies and mechanistic experimental studies. Second, future studies could identify specific T cell subsets to further estimate the TIME of HCC. The model was constructed based on TCGA cohorts, which may compromise the applicability of the conclusion in other cohorts. Finally, some risk factors of HCC patients, like AFP levels and viral hepatitis, were missing from the database, which may reduce the predictive power of the nomogram.

## 5 Conclusion

In conclusion, we constructed a novel CD4+ Tconv-related lncRNAs risk signature based on scRNA-seq data. The risk signature could reflect the TME, drug response, and prognosis of HCC patients, which could provide references for clinical practice.

## Data availability statement

Publicly available datasets were analyzed in this study. This data can be found here: https://portal.gdc.cancer.gov/; https://www.ncbi.nlm.nih.gov/geo/query/acc.cgi?acc=GSE140228.

## Author contributions

LZ, XPZ and SX conducted the analysis and wrote this manuscript. RL, LZ and XPZ designed this study. MGH, ZMZ, GDZ, ZHX contributed to manuscript writing and data analysis. All authors contributed to the article and approved the submitted version.

## Supplementary material

Supplementary Figure 1 Flowchart of the study.

Supplementary Figure 2 HCC single-cell sequencing data from the SMART-seq2 platform. **(A)** The UMAP plot of different cell types. **(B)** Ratios of different cell types.

Supplementary Figure 3 Lasso regression. **(A)** Cross-validation for tuning parameter selection in lasso regression. **(B)** Validation of tuning parameter selection by lasso regression.

Supplementary Figure 4 Time-dependent ROC curves of **(A)** training cohort, **(B)** validation cohort 1, and **(C)** validation cohort 2.

Supplementary Table 1 Baselines of patients in two validation cohorts
