# Supplementary figures and images for: Identification of a CD4+ conventional T cells-related lncRNAs signature associated with hepatocellular carcinoma prognosis, therapy, and tumor microenvironment
Source: Front Immunol. 2023 Jan 9;13:1111246. doi: 10.3389/fimmu.2022.1111246 (PMC9868629; doi:10.3389/fimmu.2022.1111246)

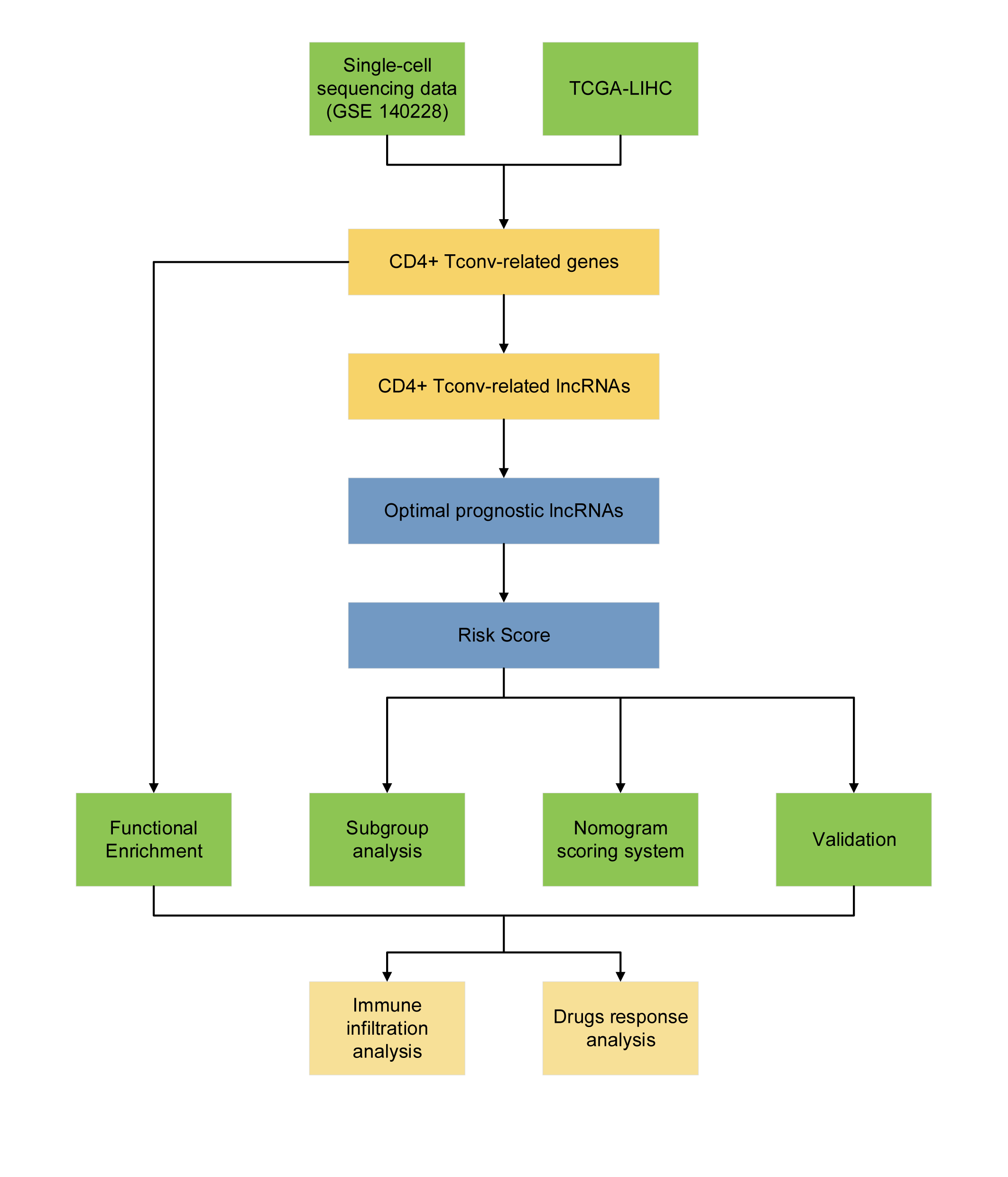

Supplement: Supplementary Figure 1 — Flowchart of the study. [file Image_1.tif]

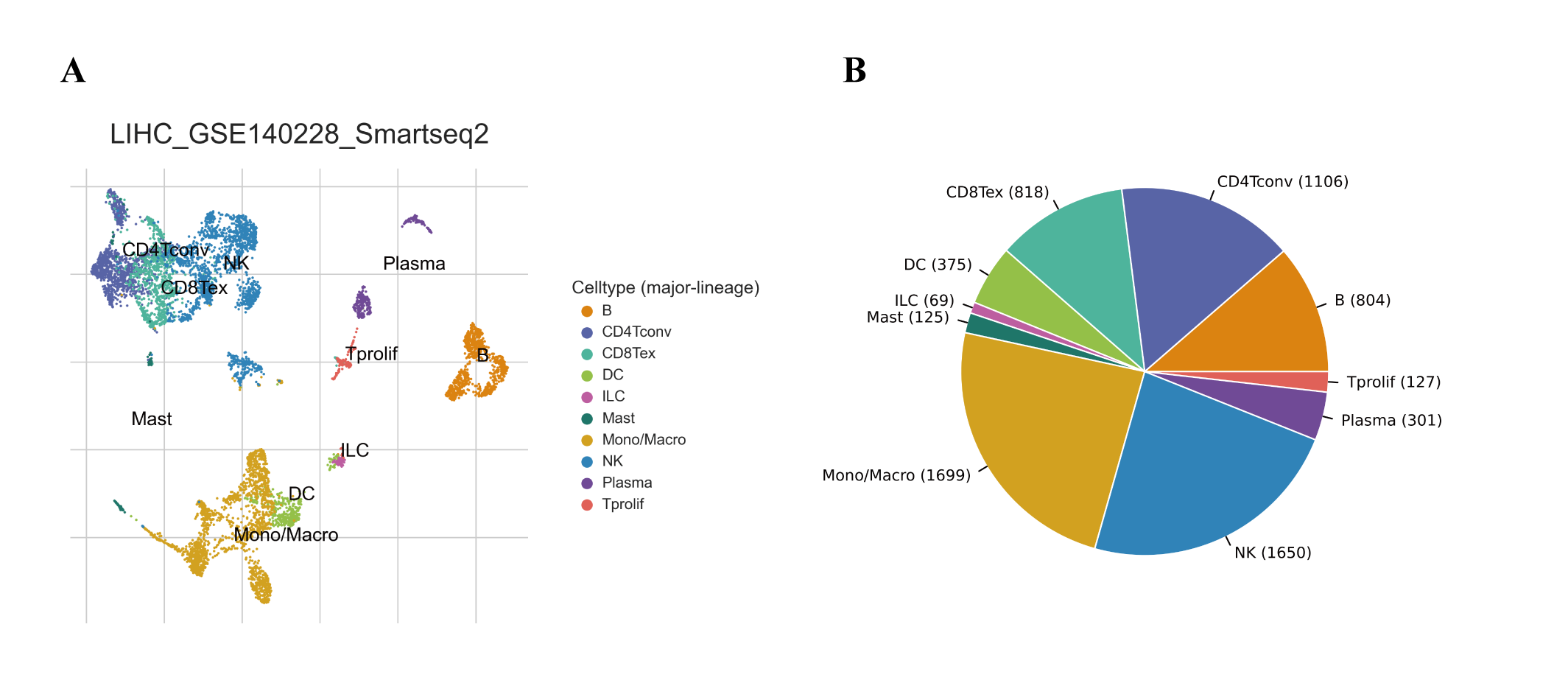

Supplement: Supplementary Figure 2 — HCC single-cell sequencing data from the SMART-seq2 platform. (A) The UMAP plot of different cell types. (B) Ratios of different cell types. [file Image_2.tif]

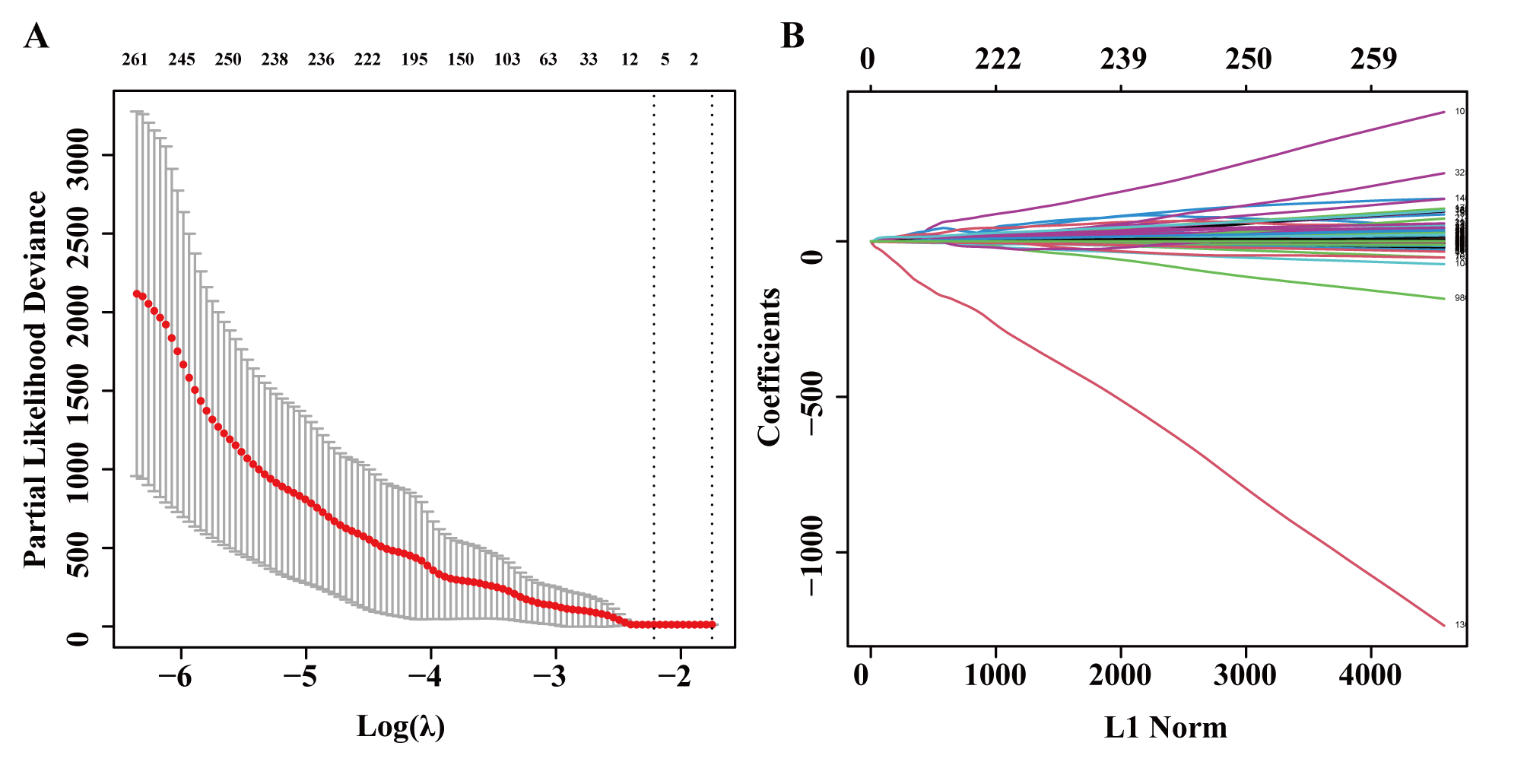

Supplement: Supplementary Figure 3 — Lasso regression. (A) Cross-validation for tuning parameter selection in lasso regression. (B) Validation of tuning parameter selection by lasso regression. [file Image_3.tif]

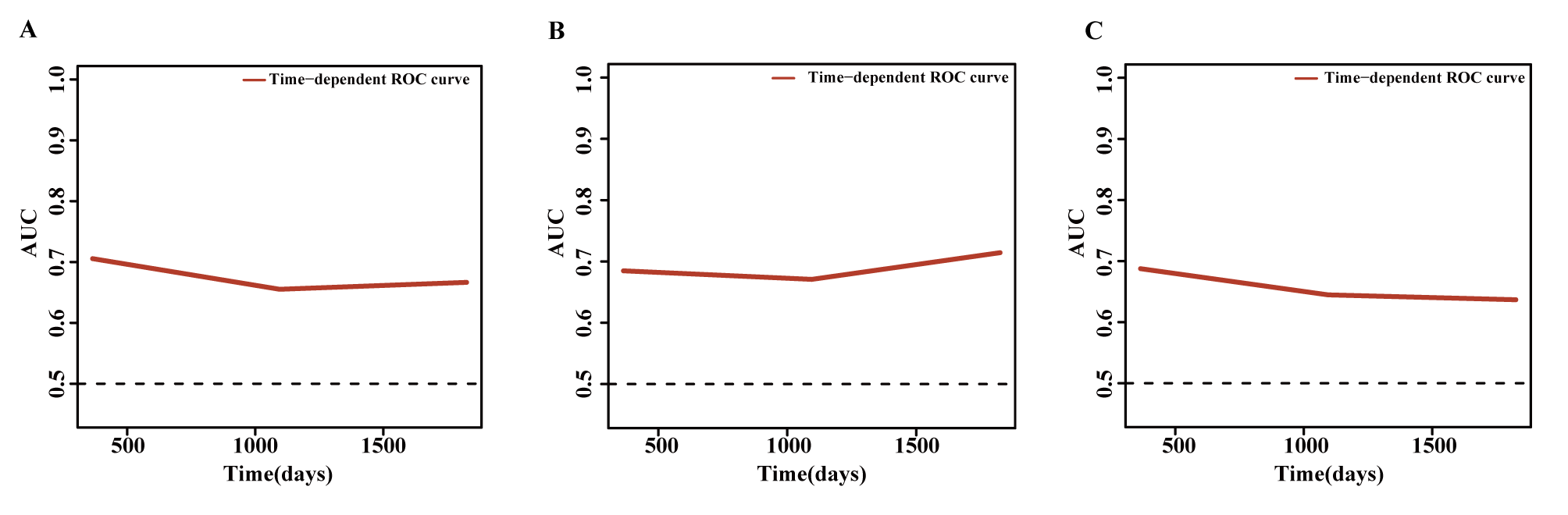

Supplement: Supplementary Figure 4 — Time-dependent ROC curves of (A) training cohort, (B) validation cohort 1, and (C) validation cohort 2. [file Image_4.tif]
