# Supplementary material for: Identification of a CD4+ conventional T cells-related lncRNAs signature associated with hepatocellular carcinoma prognosis, therapy, and tumor microenvironment
Source: Front Immunol. 2023 Jan 9;13:1111246. doi: 10.3389/fimmu.2022.1111246 (PMC9868629; doi:10.3389/fimmu.2022.1111246)
Supplement: Supplementary Table 1 — Baselines of patients in two validation cohorts [file Table_1.docx]

Supplementary Table 1. Baselines of patients in two validation cohorts.

|  | **Validation 1** | **Validation 2** | **p** |
| --- | --- | --- | --- |
|  | ***N=182*** | ***N=182*** |  |
| Age | 61 (51 to 69) | 62 (52 to 69) | 0.633 |
| Gender: |  |  | 0.094 |
| female | 67 (36.8%) | 52 (28.6%) |  |
| male | 115 (63.2%) | 130 (71.4%) |  |
| M stage: |  |  | 1.000 |
| M0 | 124 (99.2%) | 138 (98.6%) |  |
| M1 | 1 (0.80%) | 2 (1.43%) |  |
| N stage: |  |  | 0.586 |
| N0 | 120 (97.6%) | 127 (99.2%) |  |
| N1 | 3 (2.44%) | 1 (0.78%) |  |
| AJCC stage: |  |  | 0.590 |
| Stage1 | 78 (46.7%) | 91 (52.6%) |  |
| Stage2 | 41 (24.6%) | 43 (24.9%) |  |
| Stage3 | 46 (27.5%) | 37 (21.4%) |  |
| Stage4 | 2 (1.20%) | 2 (1.16%) |  |
| T stage: |  |  | 0.856 |
| T1 | 87 (48.3%) | 92 (50.8%) |  |
| T2 | 44 (24.4%) | 47 (26.0%) |  |
| T3 | 42 (23.3%) | 36 (19.9%) |  |
| T4 | 7 (3.89%) | 6 (3.31%) |  |
| Prior malignancy: |  |  | 0.471 |
| no | 163 (89.6%) | 167 (91.8%) |  |
| yes | 19 (10.4%) | 15 (8.24%) |  |
| Radiation Therapy: |  |  | 0.256 |
| No | 157 (91.8%) | 166 (94.9%) |  |
| Yes | 14 (8.19%) | 9 (5.14%) |  |
| RS | 4.99 (3.12 to 8.29) | 4.44 (3.02 to 7.80) | 0.469 |
